# Non-Humanized Vaccine Virus

**Published:** 1882-02-20

**Authors:** 


NON-HUMANIZED VACCINE VIRUS.—See advertisement of H. M. Merrell & Co., Cincinnati, O., in relation to genuine vaccine virus.

